# Activity-Based Therapy for Mobility, Function and Quality of Life after Spinal Cord Injuries—A Mixed-Methods Case Series

**DOI:** 10.3390/jcm12247588

**Published:** 2023-12-08

**Authors:** Camila Quel de Oliveira, Anita Bundy, James W. Middleton, Kathryn Refshauge, Kris Rogers, Glen M. Davis

**Affiliations:** 1 Sydney School of Health Sciences, Faculty of Medicine and Health, The University of Sydney, Sydney, NSW 2006, Australia; anita.bundy@sydney.edu.au (A.B.); kathryn.refshauge@sydney.edu.au (K.R.); glen.davis@sydney.edu.au (G.M.D.); 2 Discipline of Physiotherapy, Graduate School of Health, Faculty of Health, University of Technology Sydney, Ultimo, NSW 2007, Australia; 3 Department of Occupational Therapy, Colorado State University, Fort Collins, CO 80524, USA; 4 John Walsh Center for Rehabilitation Research, Kolling Institute, Northern Sydney Local Health District and Sydney Medical School Northern, The University of Sydney, Sydney, NSW 2006, Australia; james.middleton@sydney.edu.au; 5 Graduate School of Health, Faculty of Health, University of Technology Sydney, Ultimo, NSW 2007, Australia; kris.rogers@uts.edu.au

**Keywords:** spinal cord injuries, exercise, rehabilitation, activity-based therapies, recovery, physical activity

## Abstract

(1) Background: Despite inconclusive evidence on the benefits of activity-based therapies (ABTs) in people with spinal cord injuries, implementation has occurred in clinics worldwide in response to consumers’ requests. We explored the clinical changes and participants’ perceptions from engaging in an ABT program in the community. (2) Methods: This mixed-methods study involved a pragmatic observational multiple-baseline design and an evaluation of participants’ perceptions. Fifteen participants were included. Outcome measures were balance in sitting using the Seated Reach Distance test, mobility using the Modified Rivermead Mobility Index and quality of life using the Quality of Life Index SCI version pre- and post-participation in an ABT community-based program. Linear mixed models and logistic regressions were used to analyse the effects of intervention. Semi-structured interviews explored participants’ perceptions using inductive thematic analysis. (3) Results: There was an increase of 9% in the standardised reach distance (95% CI 2–16) for sitting balance, 1.33 points (95% CI: 0.81–1.85) in mobility and 1.9 points (0.17–2.1) in quality of life. Two themes emerged from the interviews: (1) reduced impact of disability and an increased sense of life as before, and (2) the program was superior to usual rehabilitation. No adverse events related to the intervention were observed. (4) Conclusion: ABT delivered in the community improved clinical outcomes in people with a chronic SCI. High levels of satisfaction with the program were reported.

## 1. Introduction

Over the past 30 years, evidence has grown related to spinal cord plasticity, repair and regeneration. However, in many countries, the concept of an irreparable, “hard-wired” spinal cord still guides rehabilitation after a spinal cord injury (SCI) [1]. In general, physical rehabilitation has focused solely on teaching patients how to achieve independence in day-to-day functioning by using compensatory techniques and assistive devices to overcome significant neurological deficits [2,3].

Animal studies have suggested that repeated sensory stimulation and intense exercise can elicit neuroplastic changes to the spinal cord and brain [4,5,6,7,8]. In humans, functional recovery may occur when appropriate levels of sensory stimulation associated with repetitive exercise for the areas above and below the damaged spinal cord are employed [1,9]. Neuroplasticity and recovery of function, therefore, seem to be possible in people with SCI, but how to optimise recovery and achieve clinically worthwhile changes in mobility, independence and quality of life remains unclear [9].

Exercises that target systems below the level of injury (muscle, bone and circulation) are crucial to improve health outcomes and prevent complications due to paralysis after SCI [10,11,12]. New interventions aiming not only to facilitate tasks using compensatory strategies but also focused on functional improvements through neurological recovery have been termed “Activity-Based Therapies” (ABT), and numerous programs have emerged with the aim to assist people with SCI to achieve their maximal potential for recovery [1,9,13,14].

To date, ABT interventions have been most frequently investigated for their efficacy and effectiveness using single training modalities, such as locomotor training, functional electrical stimulation (FES) or robotics [15]. Two community-based randomised controlled trials (RCTs) that compared multimodal ABT to no-intervention or self-selected exercises found greater improvements in the ABT group for neurological function and walking capacity but had inconclusive results for independence and general mobility [16,17]. In contrast, another RCT compared a 12-week multimodal ABT intervention to usual care during inpatient rehabilitation, showing no differences in neurological recovery and functional or behavioural outcomes between groups [18]. 

Previous studies have highlighted the gap between research findings and clinical practice. There is an increasing demand for evidence-based clinical practice; however, most evidence-based recommendations for clinical interventions are derived from highly controlled efficacy trials [19]. The highly controlled nature of randomised controlled trials is important to draw causal inferences; however, their focus on internal validity often reduces external relevance and generalisability, limiting implementation into clinical practice. Hence, there is a need for more studies to be conducted in settings where community constraints are prioritised over optimal conditions, including the testing of the feasibility of interventions in real-world settings [20].

Despite ambiguous evidence in support of multimodal ABT, consumer interest has encouraged many rehabilitation centres to implement ABT programs for people with SCI worldwide. However, it remains unclear what changes can be expected when ABT is provided to people with SCI living in the community, in terms of health outcomes as well as consumer satisfaction. In this study, we sought to investigate functional changes of people with SCI when following an ABT program delivered in a community setting. Hence, the objectives were: (i) to identify potential changes in mobility, functional outcomes and quality of life in people with SCI after an ABT intervention, and (ii) to examine participants’ satisfaction with ABT.

## 2. Materials and Methods

### 2.1. Study Design

We conducted an exploratory mixed-methods case series using an observational multiple-baseline single case design combined with a qualitative component. We followed participants who participated in an ABT program, using a 4-week qualification period of weekly baseline measurements to assess the stability of the primary outcomes, followed by a period of at least 4 weeks of the ABT intervention, where participants were assessed every second week. Due to the observational nature of this study, and the fact that it was conducted in a “real-world” community exercise setting, participants engaged with the program for varying frequencies and durations. Eight weeks after finishing the program, participants were reassessed to evaluate for any post-intervention changes. In addition, we conducted follow-up semi-structured interviews 1 week after the intervention period to evaluate participants’ experiences and perspectives. 

### 2.2. Participants

Participants were paying clients from the NeuroMoves ABT program, a community outreach initiative of Spinal Cord Injuries Australia (SCIA). We invited adults who had sustained SCI (at any injury level below C2 and of any lesion severity) and had contacted SCIA to enrol in the NeuroMoves ABT program at The University of Sydney, Australia, to participate. All participants were new to ABT and provided written consent following thorough client–therapist discussions. This study was approved by The University of Sydney Human Research Ethics Committee (HREC 2012/477). 

All participants needed to have a minimum total score of 2 points on the Modified Rivermead Mobility Index (MRMI) and medical permission to participate in an intensive exercise program. Participants were excluded if they were ventilator-dependent; had other associated neurological diseases; had complications such as severe urinary tract infections, pressure ulcers or osteoporosis in the lower limbs; or were diagnosed by a medical practitioner with any other health condition that could contraindicate participation in an exercise program. Participants who attended the program for a minimum of 8 weeks, were fluent in English and provided consent were asked to participate in an audio-recorded interview. 

### 2.3. ABT Program

Exercise programs were individually tailored by a physiotherapist or accredited exercise physiologist according to the person’s goals and functional abilities. The intervention involved three key elements: (i) task-specific training, (ii) weight-bearing tasks and (iii) whole-body muscle strengthening. This approach included training in different positions such as sitting on the edge of the bed, 4-point kneeling, kneeling, standing with partial or full body weight, body-weight-supported treadmill training, active-assisted exercises, resistance training, neuromuscular electrical stimulation and balance and coordination tasks. All exercises were performed out of the wheelchair, incorporating whole-body movements. (Refer to Appendix A for a detailed description of the exercise components.) Participants were encouraged to perform all exercises to their maximum capacity with 1 to 5 min for recovery, if required, between exercises. The length of intervention varied from 4 to 24 weeks with a frequency of 2 to 4 times per week. Each session was 2 h long.

### 2.4. Outcome Measures

Our primary outcomes were balance in sitting, mobility and quality of life. For balance in sitting, we used the Seated Reach Distance test (SRD) as described by Boswell-Ruys [21]. The greatest reach distance in each direction (forward, lateral left and right, diagonal left and right) was recorded in centimetres and then divided by the arm length to constitute a normalised score. Thus, the score can be interpreted as the percentage of the arm length that the individual was able to reach in each direction. A final score was obtained by calculating the mean score of all directions. The Modified Rivermead Mobility Index (MRMI) was used to assess mobility. The scoring for each task is based on a scale from 0 (unable to perform the task) to 5 (performs the task independently) and reflects the amount of assistance necessary to perform each task [22]. The Quality of Life Index (QoLI) SCI version measures satisfaction within four quality of life domains: health and functioning, psychological/spiritual, social and economic, and family. The maximum score is 30; higher scores reflect better quality of life [23,24].

Our secondary outcomes were independence during activities of daily living using the Spinal Cord Independence Measure version III (SCIM) [25], participation using the Community Integration Questionnaire (CIQ) [26], and satisfaction with life using the Satisfaction with Life Scale (SWLS) [27]. Adverse events, defined as any untoward medical occurrence that does not necessarily have a causal relationship with the intervention, were monitored and recorded [28]. During the multiple-baseline 4-week period, we assessed the primary outcomes weekly (same day of the week and time of day). After completing the multiple-baseline assessment period, we evaluated participants at the start and end of the intervention period, and at 8-week follow-up for primary and secondary outcomes. In addition, the primary outcomes were assessed at 2-week intervals during the intervention period.

### 2.5. Interviews

We conducted a semi-structured interview (Appendix B) with individuals who completed at least 8 weeks of the ABT intervention in the week of or 1 week following the participants’ final assessment to capture their overall perceptions of, and satisfaction with, the program. The interview included questions about participants’ opinions and beliefs about ABT, any perceived changes resulting from the program and the logistics involved in participating in the ABT exercise program. All questions were open-ended; follow-up probes were used to gain a more in-depth understanding of participants’ responses. Recordings of the interviews were transcribed verbatim by a professional transcriber. As part of the interview, participants also rated aspects of the program on a scale from 0 to 10, with 0 indicating “really bad” and 10 being “really great”. 

### 2.6. Data Analysis

Only participants who were exposed to the intervention were included in the data analysis. Statistical analyses of the primary outcomes were conducted independently of the primary author by a professional biostatistician who was otherwise not involved in the study (KRo). The analysis for the primary-outcome measures was conducted using R software version 4.1 packages lme4 and nlme [29]. For other analyses, the software IBM SPSS Statistics 22™ was utilised.

Primary-outcome measures: Linear mixed-model regression was used to assess changes in outcomes across baseline and intervention phases. We fitted a model with a random intercept for each participant (allowing for each participant to have a unique baseline starting measurement), and with (a) time included as a fixed effect (i.e., there was a common trajectory of the outcomes over time) and (b) time included as a random effect (i.e., each patient had their own trajectory in how the outcomes changed over time). We assessed whether there was an overall or individual trajectory by assessing measures of model fit using Akaike’s Information Criteria (AIC) and Bayesian Information Criteria (BIC) and visually inspecting the predicted outcomes for each participant over time against the actual values. 

Secondary-outcome measures: The effects of treatment on the secondary-outcome measures were assessed by paired-sample *t*-tests at the 5% confidence level. 

Maintenance of effects: We obtained 8-week follow-up assessments for only 4 of the 13 participants. Due to the small sample size, the follow-up period was not included in the statistical analysis and data are presented individually. 

### 2.7. Qualitative Analysis

Inductive thematic analysis of semi-structured interviews was used, as described by Braun and Clarke [30]. An open coding process was performed whereby all passages in each interview were examined line by line and coded to reflect the content. One of the interviews, judged by the first author to be particularly difficult to code, was reviewed in its entirety by a more experienced author (AB) and discussed until consensus was reached on the coding. The first author then recoded all the interviews, and the recoding was reviewed by the experienced author. 

## 3. Results

### 3.1. Participants

**Figure 1:**
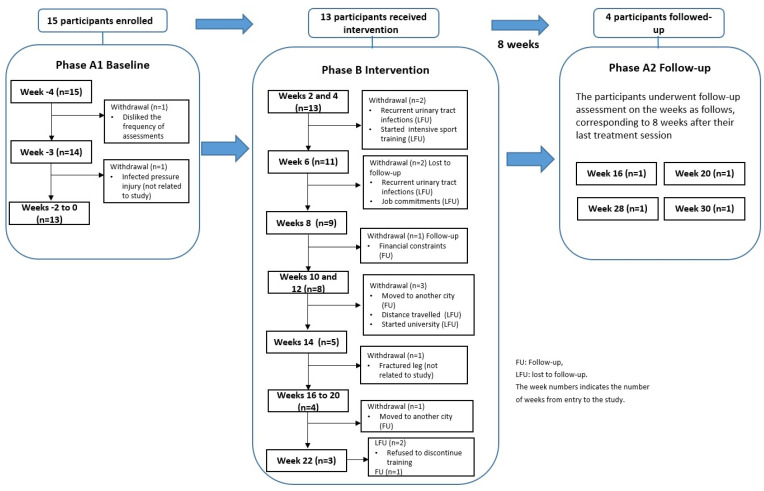
Flow of participants through the study with reasons for dropouts.

Fifteen NeuroMoves clients expressed interest in participating in the study after contact from an SCIA staff member (not involved in the study) and met the inclusion criteria. All 15 participants completed a weekly assessment during 4 weeks of multiple-baseline data collection. Thirteen participants completed the intervention period, two having withdrawn for medical reasons or to engage in sports activities. Eight participants undertook the semi-structured interview. The flow of participants through the study, with reasons for dropping out, is shown in Figure 1.

The characteristics of the 13 participants who completed the study and duration of their intervention are described in Table 1. The mean age of participants was 32.1 years (SD = 12.4), and the mean duration post-injury was 46.5 months (SD = 65.9).

We attempted to fit both random intercept and random intercept + slope models to all variables but were only able to do so for the quality of life outcome. Both measures of model fit indicated that the random-intercept-only model had the best fit. For MRMI and SRD, the models including a random effect of time did not converge, which indicated that they were a poor fit to the data. We, therefore, considered random intercept models with an overall trajectory for the three outcomes. See Appendix C. Table A1.

### 3.2. Sitting Balance

**Figure 2:**
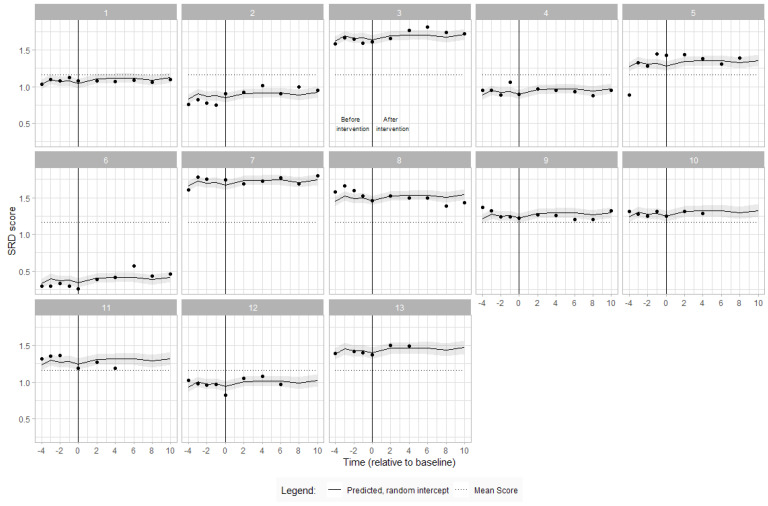
Seated Reach Distance (SRD) scores over time for each participant with estimated SRD from the linear mixed model and overall mean score. The participant number is indicated at the top of each graph. The vertical line denotes commencement of ABT intervention. Each dot represents an assessment timepoint.

Visual inspection of the data reveals high variability on SRD scores at baseline. Overall, participants had similar SRD across baseline, and with good evidence of an increase of 0.09 (95% CI 0.02–0.16), meaning that participants could reach a further 9% of their arm length in sitting by the end of the intervention period. Figure 2 shows the individual SRD scores during the baseline and intervention periods.

### 3.3. Mobility

**Figure 3:**
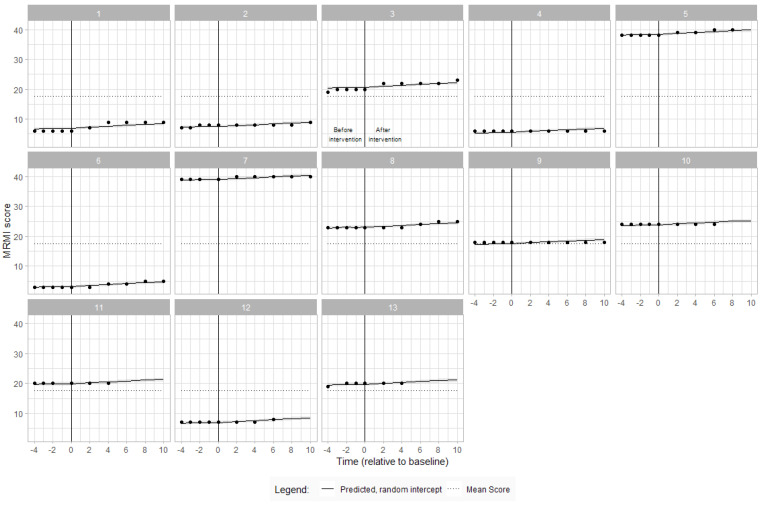
Modified Rivermead Mobility Index (MRMI) scores over time for each participant with estimated MRMI from the linear mixed model and overall mean score. The participant number is indicated at the top of each graph. The vertical line denotes commencement of ABT intervention. Each dot represents an assessment timepoint.

Visual inspection of the data demonstrates stability of scores during the baseline period for most participants, except for participants 3 and 13, who exhibited a slight positive slope during the baseline phase. There was a small but inconsistent shift in MRMI before the intervention period of 0.23 (95% CI: −0.22–0.68), and then the overall trajectory was for MRMI to steadily increase by 1.33 (95% CI: 0.81–1.85) points after intervention. There were no changes in mobility for participants 4, 9, 10 and 11 when compared to baseline. Figure 3 portrays the individual MRMI scores during the baseline and intervention periods.

### 3.4. Quality of Life

**Figure 4:**
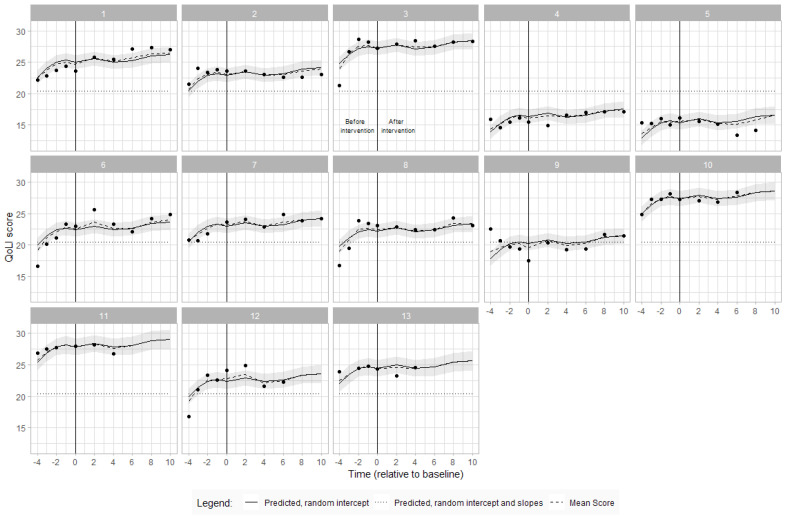
Quality of Life Index (QoLI) scores over time for each participant with estimated QoLI scores from the linear mixed model and overall mean score. The participant number is indicated at the top of each graph. The vertical line denotes commencement of ABT intervention. Each dot represents an assessment timepoint.

Visual inspection demonstrates considerable variability in the quality of life scores at baseline. There was an overall increase in slope of 2.42 points (95% CI: 1.35–3.50) during baseline, and a smaller increase of 1.9 points (95% CI:0.17–2.1) by the end of the intervention phase. For most participants, the slope was positive during baseline, followed by an increase of small magnitude during the treatment period. The exception was participant 9, who showed a steep decline in quality of life during baseline followed by a slight positive change during the intervention period. Participant 5 also presented a negative change during intervention when compared to baseline. Figure 4 shows the individual quality of life scores during the baseline and intervention periods. 

### 3.5. Secondary Outcomes

There were small positive changes in the SCIM with a mean change in score of 2.2 (95% CI: 0.3 to 4.2) points and in the CIQ of 1.7 (95% CI 0.3 to 3.1) points after participation in the ABT program. No significant changes were seen in the SWLS with a mean change in score of 2.0 (95% CI: −0.5 to 4.4) points, with the 95% confidence interval spanning zero (Appendix D).

### 3.6. Retention and Adherence

Attendance varied in duration from 4 to 22 weeks (average 12.6, SD = 6.94), with most participants (11/13) attending two sessions per week. Only four participants undertook the 8-week follow-up assessment. Most participants left the program due to health-related problems, such as urinary tract infections. Engagement in social activities such as sports, work, university and moving to another city were also reasons to discontinue participation. The reasons for loss to follow-up are presented in Figure 1. 

### 3.7. Maintenance of Effects

Participants 1, 2, 5 and 7 underwent follow-up assessments. There was a further small improvement in quality of life of 1% and 6% for subjects 1 and 7. Participant 5 had no changes in quality of life at follow-up, while participant 2 declined by 7%. The changes in mobility were maintained for all participants. There was some stability in balance in sitting, with two participants (1 and 5) maintaining the changes and two participants (2 and 7) experiencing a slight decrease in the MRMI at follow-up of 4% and 5%, respectively. All participants continued to show some improvements in independence that ranged from 2% to 11%. Community integration continued to improve for participants 1 and 5 after cessation of intervention, by 7% and 18%, respectively, although the score returned to the baseline value for participant 7. Supplemental follow-up data for each subject is contained in Appendix E.

### 3.8. Adverse Events

No adverse events related to the intervention were observed. One participant discontinued involvement in the study during the baseline period due to infected pressure injuries, and three discontinued during the intervention period: two due to recurrent urinary tract infections and one due to a leg fracture.

### 3.9. Participants’ Perceptions and Experiences

Analysis of the content of the interviews (n = 8) yielded two themes: (a) the impact that my disability has in my life has decreased (i.e., sense of life as before), and (b) the program is different from (superior to) usual rehabilitation. These themes revealed that all participants believed the program was beneficial and yielded physical and psychosocial gains. A summary of the themes and subthemes generated by the thematic analysis appears in Table 2.

#### 3.9.1. Theme 1: The Impact That My Disability Has in My Life Has Decreased (Sense of Life as before)

Overall, participants enjoyed attending the program and reported that the positive experiences outweighed any negative aspects. Seven participants rated the program with a score of at least 8 out of 10; the eighth participant rated it between 6 and 7. All participants acknowledged the need for and importance of community-based rehabilitation programs; the majority indicated they would recommend it to anyone with an SCI. This general perspective is reflected in the following quotes:

*“The improvement is just unbelievable. So, for me, that’s the best thing about it, and the bad thing about it is I can’t do it more times than I do. I really enjoy it.”*

*(P8)*

*“Before I wouldn’t have gone out of the golf club, now I go out to the golf course and walk around a bit, and even around the restaurant after having dinner and a few drinks and staying around rather than just not doing any social activities at all.”*

*(P5)*

All participants reported psychological and emotional gains, such as feeling more motivated and happier. Participation in the program led to increased confidence associated with greater independence and a willingness to attempt tasks and be involved in activities that they had previously considered impossible due to the injury.

*“It’s not just the functional benefits. It’s the psychological and emotional positives that come out of it. Feeling more confident and feeling better, having more independence. I think that’s what most people want to gain after they’ve had an injury.”*

*(P2)*

Combined physical and psychological gains made participants’ everyday lives easier with less reliance on others for assistance or use of external aids and functioning more like before their injury.

*“I’m eating with whatever utensils I have. I can actually remove them from my lap to the table without any assistance... I can grab the remote, work the remote from the shelf in my room onto my lap to access the TV if I want…I found eating most types of food that I’ve had trouble with beforehand have been a lot easier, like holding something like a burger.”*

*(P1)*

Participants reported that the program gave them access to a new community by providing an environment where they could socialise with others in similar situations. One participant compared it to being part of a sports team. Participants regained the opportunity to connect and socialise with a variety of people and not be treated as “*different*” or “*disabled*”. Talking to people in a similar situation allowed them to learn from each other and exchange experiences that helped to deal better with their injury.

#### 3.9.2. Theme 2: The Program Is Different from (Superior to) Usual Rehabilitation

Participants used words to describe the program such as “*fun*”, “*unique*”, “*novel*” and “*exciting*” when making comparisons to the hospital system and conventional physiotherapy programs. They evaluated the ABT program as being superior due to the variety of equipment and exercises available, and the uplifting and encouraging environment. 

Participants considered the exercise modalities and equipment used during sessions as novel and unique. Exercising out of the wheelchair in different body positions, including standing, was considered vital for their health and functional recovery. Even if their goals did not include standing or walking, being in the upright position and having the lower limbs stimulated to move seemed to have positive physical and psychological effects.

*“For me, is all about getting out of my chair. When you are in the chair you are mainly just restricted to doing weights, maybe a bit of trunk. When you get out of the chair at least you can stretch your whole body. You use everything. You try different exercises. You not only work out what you have but just test out and try and work out things that are weaker or that you don’t have.” *

*(P2)*

Negative aspects of the program were reported; however, participants qualified that these were relatively minor. The distance travelled to get to the program was perceived as a negative. The time commitment and the cost were considered high. However, most participants considered the cost and time invested worthwhile, given the benefits. Other negative aspects were the different levels of skill and expertise amongst staff and the size of the exercise area, which sometimes became too busy, interfering with the quality of the sessions and making performance of certain exercises and the use of certain equipment impractical. 

## 4. Discussion

To our knowledge this is the first multiple-baseline study to assess the potential effects of ABT in a “real-life” setting, while adding contextual details about participants’ perceptions and experiences. Overall, we found that ABT led to small positive changes in sitting balance, mobility and quality of life in people with a chronic SCI. Furthermore, the qualitative analysis showed high levels of satisfaction and perceived benefits that surpassed what was identified by the outcome measures. However, the magnitude of change was small and possibly not clinically meaningful. The SCIM III was the only outcome measured in the current study that has previously been found to have a minimal clinically important difference in a population with SCI [31]. An increase of at least four points on the total SCIM during acute care/subacute rehabilitation is usually considered to equate to a small but worthwhile improvement, and an increase of 10 points to equate to a substantial improvement [32].

All participants in our study had undergone in-hospital rehabilitation programs and had been injured for at least six months. Therefore, they were not expected to experience further changes in their functional status due to the reduced potential for spontaneous recovery, especially for motor function, wherein the greatest rate of change occurs within the first three months post-injury [33]. Furthermore, most participants in our sample (n = 11/13) had a motor-complete injury (AIS A and B), which might have contributed to the small magnitude of the gains. Previous research [34,35,36] showed that individuals with motor-incomplete injuries (AIS C and D) experience greater benefits from ABT than individuals with motor-complete injuries (AIS A and B). 

Regardless of the small changes in the outcome measures employed in this study, the perceived benefits reported by the participants were in many areas including independence and community participation, which corroborates a more recent qualitative study where ABT participants reported a perceived positive impact on physical, functional and psychosocial domains, leading to improved independence and quality of life. Furthermore, their participants believed that ABT had a key role in the evolving and lifelong recovery after SCI [37]. Overall, the analysis from the interviews showed that the perceived benefits were greater than the changes detected by the outcome measures. The discordance between patient-reported and performance-based tools could be due to an overestimation of the changes by the participants, which could be related to the (1) novelty effect, (2) lower expectation of rehabilitation outcome and (3) Hawthorne effect. Firstly, the novelty effect is defined as “the perceived usability of something on the account of newness” [38]. In our study, all participants were new to ABT, which could have resulted in a bias towards overestimation of benefits due to excitement with the opportunity to access the novel treatment. Secondly, the level of expectation could have affected the participants’ perception of change. The lack of improvements experienced in the chronic phase after SCI could have accounted for a lowered expectation for recovery after that time and therefore resulted in an overestimation of perceived improvement, which means that even a subtle change will be perceived as important and significant [39]. Thirdly, the Hawthorne effect is a phenomenon where participants tend to report the effects of a treatment differently due to being aware of the nature and purpose of the study [40]. Our participants were informed about the nature and purpose of the study prior to enrolment, which could have led them to providing more positive perspectives and opinions about the training they received.

Most participants attended only two 2-h sessions per week, which could have contributed to the relatively small magnitude of the improvements identified by the standardised outcome measures. Other researchers have reported that ABT performed for 2 to 5 h per day at a frequency of three to five times per week produced benefits in participants with chronic SCI that included improved lower-limb muscle strength, balance, mobility, increased gait speed, symmetry and endurance [2,41,42]. Only two participants attended more than two sessions per week. When observing the individual data, the participants who attended the program at a higher frequency experienced greater improvements than individuals with similar injury characteristics who attended fewer sessions.

Previous research focused on assessing the effects of ABT interventions on outcomes related to lower limb mobility, such as walking, standing and balance [15,43,44,45,46]. Typically, ABT involves the practice of intense exercise, performed out of the wheelchair, in various body positions, mostly against gravity, which can positively impact other aspects of mobility and function, as well as enhance psychological outcomes, such as mood and self-image, promoting greater quality of life [47,48,49,50]. The fact that the ABT intervention in our study was delivered in a community setting is likely to have promoted social interactions that were beneficial for the participants’ mental health, as identified in the interviews. Hence, it is important to investigate not only the effects of a multimodal ABT exercise program on functional outcomes related to physical abilities, but also its impact on psychological well-being, quality of life and community participation. Furthermore, due to the growing numbers of people with SCI seeking to participate in ABT programs in the community [51], it is necessary to assess this intervention when applied outside the research setting.

Cost, distance and time commitment are possible barriers for participation in ABT and should be considered when implementing ABT programs in the community, particularly given that evidence shows that high intensity and frequency seem to be determinants for the effectiveness of ABT [2,52,53,54]. Our findings support the necessity of ABT programs in the community to be delivered at high frequency per week for greatest benefits. One strategy to overcome the barriers while delivering an effective dose of ABT may be to deliver it in a block of high-frequency for short periods. Such an approach may facilitate adherence and reduce disruption of other community engagement activities. 

### Study Limitations

In this study, we evaluated the changes and participants’ perceptions of a multimodal ABT intervention in a clinical setting that reflected the reality of a community-based clinic. Several limitations affect the generalisation of our findings, such as the clinical heterogeneity of participants, different lengths of exposure to the intervention, the fact that participants were paying to attend the program, loss to follow-up and the small sample size. We believe that the small sample size was driven largely by potential participants not being willing to wait for the four-week baseline assessments before starting the program. The high degree of variability in the number of weeks that the intervention was delivered was potentially caused by the reduced ability of participants to attend a long-term ABT program due to costs, distance to travel and difficulties fitting it in with other life demands. 

Another limitation was the lack of stability of baseline measures for quality of life and Seated Reach Distance. Studies employing a multiple-baseline design can demonstrate if a significant change in behaviour has occurred as result of the intervention. However, strong conclusions can only be drawn when the baseline is neutral or in the opposite direction to an observed behaviour [55,56]. 

We were unable to assess maintenance of the changes from the ABT treatment, mainly due to the high number of participants lost to follow-up. Despite considerable variability in outcomes at the follow-up assessment across remaining participants, some maintenance of mobility was observed. Two of the three participants who completed the maximum intervention period of 22 weeks were unwilling to discontinue the ABT treatment due to concerns that an 8-week pause would cause deterioration, delay or hinder further progress in important benefits already achieved. These participants were free to continue to attend the NeuroMoves ABT program; thus, duration of outcomes could not be measured. 

Lastly, the high satisfaction levels and perceived changes reported by participants in the present study may have been influenced by the fact that they enrolled in the ABT program independently, leading to participation bias. They were potentially more motivated, open to new interventions and have more financial resources than the general SCI population, potentially biasing the findings.

## 5. Conclusions

ABT delivered in the community can be beneficial and was well regarded by participants with a chronic SCI. Multimodal ABT programs, applied after in-patient rehabilitation, can maximise gains in the outcomes of quality of life, mobility, sitting balance, independence and community participation. However, the changes were of small magnitude, possibly due to the challenges in achieving the recommended therapeutic dosage in a community setting.

## Figures and Tables

**Table 1:** Participants characteristics and duration of intervention.

Participant	Age (Years)	Gender	Duration Post-Injury (mo)	Level of Injury	AIS Classification	Number of Sessions Attended	Number of Sessions per Week
***1***	***22***	***M***	***26***	***C5***	***A***	***43***	***4***
***2***	***23***	***F***	***10***	***C5***	***A***	***45***	***2***
***3***	***37***	***F***	***6***	***T11***	***C***	***46***	***2***
***4***	***20***	***M***	***11***	***C4***	***B***	***42***	***2***
***5***	***54***	***M***	***15***	***C3***	***D***	***16***	***2***
***6***	***31***	***M***	***12***	***C4***	***B***	***70***	***3***
***7***	***20***	***F***	***6***	***L1***	***A***	***52***	***2***
***8***	***43***	***M***	***30***	***T4***	***B***	***45***	***2***
9	23	F	212	T2	A	15	2
10	33	M	121	T12	B	14	2
11	56	M	135	T6	A	11	2
12	32	M	9	C5	B	19	2
13	23	M	12	T11	A	15	2

M: male, F: female; AIS: ASIA Impairment Scale; A: complete; B: sensory incomplete; C: motor incomplete (more than half of key muscle functions below level of injury have a muscle grade less than 3); D: motor incomplete (at least half of key muscle functions below level of injury have a muscle grade greater than 3). Participants in bold italic have completed the qualitative arm of this study.

**Table 2:** Themes and subthemes identified in the inductive thematic analysis.

**1.** **The impact that my disability has in my life has decreased (sense of life as before).**
1.1 I am more independent and participate in life again. 1.2 I am more confident to do things by myself and to attempt new things. 1.3 The gains that I got from the program made my everyday life easier. 1.4 My improvements had a positive impact on my family and friends. 1.5 I feel part of a community again.
**2.** **The program is different from usual rehabilitation**
2.1 I tried new things (exercises and equipment) including exercising out of the wheelchair. 2.2 The environment is uplifting and motivating. 2.3 The treatment is individually tailored and specialised to my injury and needs. 2.4 The amount of time and money invested was big, but it was worth it. 2.5 Negatives, of course, but they are minor.

## Data Availability

Data are available upon contact with the corresponding author, Dr Camila Quel de Oliveira. Email: camila.queldeoliveira@uts.edu.au.

## Appendix A. Key Elements of Multimodal ABT

- Active-assisted exercises: Performed on a plinth or mat in supine, prone or side-lying position. The therapist assisted the lower-limb movements through different ranges of motion, and when possible provided resistance. Participants were encouraged to attempt and visualise actively assisting or resisting the movement performed.
- Resistance training for upper and/or lower limbs: Used when a participant demonstrated voluntary motor control and consisted of concentric and eccentric exercises against gravity or adding external resistance, such as weights or resistance bands.
- Load bearing: Consists of activities on hands or elbows and/or feet or knees in contact with the ground, with some percentage of body weight supported through the extremities. While on hands and knees or in the kneeling position, the participant is encouraged to perform upper- and lower-body movements, in order to enhance trunk and pelvic control. Crawling and locomotion in the kneeling position were also performed.
- Standing with or without standing frames: Participants stand with the assistance of a frame and are encouraged to perform trunk and arm movements, or if able, to raise themselves into a standing position to load the lower extremities, using the parallel bars, and perform lower-limb movements, until fatigue.
- Partial body weight using antigravity board: Participants complete active and/or active-assisted squat exercises (unilateral or bilateral) while partially loading their lower extremities. Participants also perform exercises for postural control in a seated position with partial body weight borne through their lower limbs.
- Leg ergometry: Using a stationary exercise bicycle, the participants are seated with trunk support provided by a therapist, if necessary, and attempt to pedal the bike under his/her own power. If unable to do so, manual external assistance is provided to complete the movement.
- Electrically stimulated leg ergometry: While seated in their wheelchair or in a chair with back support, the gluteus maximus, hamstrings, quadriceps, triceps surae and tibialis anterior could be electrically stimulated by surface electrodes, according to the participant’s needs to produce a cycling movement. Leg ergometry could also be conducted in a standing position with electrodes on the gluteus maximus, hamstrings, gastrocnemius, quadriceps and the tibialis anterior muscle groups with body-weight support and assistance of a robotic stepping device that simulates gait movements.
- Gait training or supported ambulation: Involves different forms of gait training, including body-weight-supported treadmill training with or without FES or manual external assistance, overground training with a frame with or without manual external assistance, or treadmill training without support, according to participants’ locomotor capability. These activities sometimes required assistance from up to four therapists, depending on the participant’s ability to control their trunk and lower limbs.
- Vibration training: The aim of this intervention is to promote sensory input putatively to alter muscle spindle sensitivity to stretch and modulate reflex alpha motoneuron activation of muscle contractions. Exercises are performed with the feet in contact with an oscillatory platform in either a seated or standing position.
- Task-specific training: According to the participant’s goals and abilities, this involves bed mobility, transfers, balance in sitting or standing, walking mobility and postural changes.

## Appendix B. Semi-Structured Interview

(1) Tell me about your experiences during the period that you were in the ABT program?
  - I want to know the good things and the things that weren’t good as well.
(2) Tell me about the benefits (changes) that you’ve got from the program?
  - Physically/Functionally?
  - Socially?
  - Emotionally/Psychologically/Well being?
  - Family/carers?
(3) What is your opinion about the program? Could you tell me on a scale from 0–10 your opinion about the program, being 0: really bad and 10: really great.
(4) What are your feelings about the program? How do you feel about it?
(5) Tell me about the logistics during the period that you were in the program?
  - Time
  - Transport
(6) Is there anything else that you would like to tell me?

## Appendix C

**Table A1:** Model fit parameters (Akaike Information Criterion (AIC) and Bayesian Information Criterion (BIC)) for the main study outcomes (Quality of Life Index, SCI version (QoL), Modified Rivermead Mobility Index (MRMI) and Seated Reach Distance test (SRD)) with random intercept and random intercept and slope (time as random effect) models.

Outcome	Model	AIC	BIC
QoL	Random Intercept	475.8	476.9
	Random Intercept + Slope	477.8	479.5
MRMI	Random Intercept	325.3	326.4
	Random Intercept + Slope	*	*
SRD	Random Intercept	−154.3	−153.2
	Random Intercept + Slope	*	*

* Not estimable—model failed to converge.

## Appendix D. Secondary Outcomes

**Table A2:** Changes in secondary-outcome measures.

Outcome Measure	Baseline Mean ± SD	Post-Intervention Mean ± SD	Change Score Mean ± SD	95% CI	t Value	*p*-Value	Effect Sizes (Cohen’s d)
**SCIM**	46.7 ± 24.7	48.9 ± 25.9	2.2 ± 3.2	0.3 to 4.2	2.52	0.027 *	0.09 (very small)
**CIQ**	16.5 ± 3.9	18.2 ± 4.6	1.7 ± 2.3	0.3 to 3.1	2.62	0.023 *	0.41 (small)
**SWLS**	18.3 ± 4.9	20.3 ± 7.5	2 ± 4.1	−0.5 to 4.4	1.78	0.101	-

SCIM = Spinal Cord Independence Measure version III; CIQ = Community Integration Questionnaire; SWLS = Satisfaction With Life Scale; * denotes statistical significance.

## Appendix E. Changes at Follow-Up

**Table A3:** Changes over time for the four participants that underwent all scheduled follow-up assessments.

Outcome Measures	Baseline	Post-Intervention	% Change from Baseline to Post-Intervention	Follow-Up	% Change from Post-Intervention to Follow-Up
***Participant1***					
**QoLI**	23.3 ± 0.8	26.9	16	27.2	1
**MRMI**	6.8 ± 0.4	9.0	32	9.0	0
**SRD**	1.1 ± 0.0	1.2	10	1.2	1
**SCIM**	26.0	27.0	4	30	11
**CIQ**	14.5	15.8	9	18.6	18
**SWLS**	24.0	28.0	17	24.0	−14
***Participant 2***					
**QoLI**	23.3 ± 1.0	22.8	−2	21.3	−7
**MRMI**	7.6 ± 0.6	9.0	18	9.0	0
**SRD**	0.8 ± 0.1	1.0	23	0.9	−4
**SCIM**	25.0	28.0	12	30.0	7
**CIQ**	22.0	22.0	0	22.0	0
**SWLS**	19.0	23.0	21	17.0	26
***Participant 5***					
**QoLI**	16.6 ± 0.5	14.2	−15	14.2	0
**MRMI**	38 ± 0.0	40	5	40.0	0
**SRD**	1.3 ± 0.2	1.4	11	1.4	0
**SCIM**	71.0	73.0	3	79.0	8
**CIQ**	11.3	15.0	33	16.0	7
**SWLS**	17.0	12.0	−29	13.0	8
***Participant 7***					
**QoLI**	21.7 ± 1.4	23.3	8	24.7	6
**MRMI**	39.0 ± 0.0	40.0	3	40.0	0
**SRD**	1.7 ± 0.1	1.8	2	1.7	−5
**SCIM**	81.0	92.0	14	94.0	2
**CIQ**	16.4	22.0	34	16.5	−25
**SWLS**	26.0	28.0	8	29.0	4
